# Unsupervised Domain Adaptation for Image Classification and Object Detection Using Guided Transfer Learning Approach and JS Divergence

**DOI:** 10.3390/s23094436

**Published:** 2023-04-30

**Authors:** Parth Goel, Amit Ganatra

**Affiliations:** 1 Computer Science & Engineering Department, Devang Patel Institute of Advance Technology and Research (DEPSTAR), Faculty of Technology & Engineering, Charotar University of Science and Technology (CHARUSAT), Changa 388421, Anand, India; 2 U & P U Patel Department of Computer Engineering, Chandubhai S. Patel Institute of Technology (CSPIT), Faculty of Technology & Engineering, Charotar University of Science and Technology (CHARUSAT), Changa 388421, Anand, India; 3 Computer Science and Engineering Department, Faculty of Engineering & Technology, Parul University (PU), Waghodia 391760, Vadodara, India; provost@paruluniversity.ac.in; 4 Faculty of Technology & Engineering, Charotar University of Science and Technology (CHARUSAT), Changa 388421, Anand, India

**Keywords:** domain adaptation, transfer learning, image classification, object detection, convolutional neural network, deep learning

## Abstract

Unsupervised domain adaptation (UDA) is a transfer learning technique utilized in deep learning. UDA aims to reduce the distribution gap between labeled source and unlabeled target domains by adapting a model through fine-tuning. Typically, UDA approaches assume the same categories in both domains. The effectiveness of transfer learning depends on the degree of similarity between the domains, which determines an efficient fine-tuning strategy. Furthermore, domain-specific tasks generally perform well when the feature distributions of the domains are similar. However, utilizing a trained source model directly in the target domain may not generalize effectively due to domain shift. Domain shift can be caused by intra-class variations, camera sensor variations, background variations, and geographical changes. To address these issues, we design an efficient unsupervised domain adaptation network for image classification and object detection that can learn transferable feature representations and reduce the domain shift problem in a unified network. We propose the guided transfer learning approach to select the layers for fine-tuning the model, which enhances feature transferability and utilizes the JS-Divergence to minimize the domain discrepancy between the domains. We evaluate our proposed approaches using multiple benchmark datasets. Our domain adaptive image classification approach achieves 93.2% accuracy on the Office-31 dataset and 75.3% accuracy on the Office-Home dataset. In addition, our domain adaptive object detection approach achieves 51.1% mAP on the Foggy Cityscapes dataset and 72.7% mAP on the Indian Vehicle dataset. We conduct extensive experiments and ablation studies to demonstrate the effectiveness and efficiency of our work. Experimental results also show that our work significantly outperforms the existing methods.

## 1. Introduction

Deep learning has achieved significant success in the field of computer vision in recent years, particularly in image classification and object detection using Convolutional Neural Networks (CNNs). Typically, CNNs are trained with supervised learning using large amounts of labeled data, drawn from an identical distribution for both training and testing the model. However, collecting and labeling data can be very time-consuming, labor-intensive, and expensive, especially for new tasks in various domains. In addition, adequate training samples do not always exist. Furthermore, the training of deep CNNs is domain-specific. The existing models show promising results on the dataset used for training. However, they often fail to generalize well to new, similar domains due to the problem of domain shift [1]. Domain shift arises when the distribution of data in the target domain differs from the source domain, posing a significant challenge for image classification and object detection tasks. This discrepancy can occur due to variations in the visual appearance of the data, which leads to several practical implications for real-world applications in the field of image classification and object detection. For instance, consider an intelligent system to detect objects on the road using CCTV (Closed-Circuit Television) footage captured from various camera sensors. If the training data from each of the camera sensors do not encompass variations in noise characteristics, image resolutions, and different weather conditions, the system’s performance may degrade in the presence of adverse conditions. Similarly, if we train an image classification model on data downloaded from e-commerce websites and test it on real camera images, the model’s performance is likely to be compromised due to differences in image characteristics across different domains, such as intra-class variations, camera angles, lighting conditions, and complex backgrounds. Therefore, there is a need to develop algorithms that can address both label scarcity and domain shift problems. The objective of domain adaptation approaches is to overcome these challenges by learning domain-invariant features to align the data distributions of the source and target domains.

**Figure 1:**
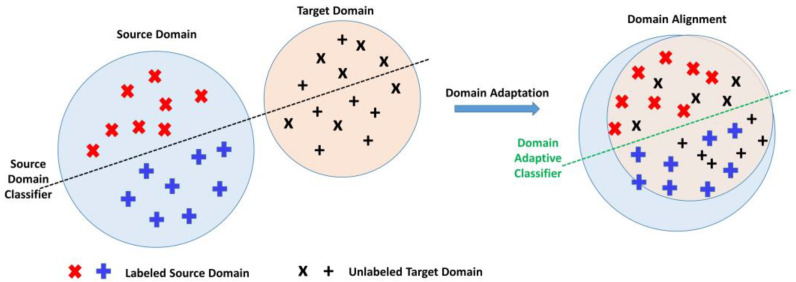
Example of unsupervised domain adaptation; Source domain and the target domain (**left**) are classified through a source-only classifier with source labeled data and target unlabeled data; Source and target domain (**right**) are classified after domain adaptation, which aligns the feature distributions of both domains.

Domain adaptation is a type of transfer learning utilized to train a model with unseen data in the target domain by acquiring knowledge from a related source domain [2]. The source domain refers to the data distribution used to train the model with labeled data for the source task, while the target domain refers to data from another related domain used to fine-tune the pre-trained model to learn the target task. There are three types of domain adaptation approaches: supervised, semi-supervised, and unsupervised [3]. Significant progress has been seen in supervised and semi-supervised domain adaptation, while unsupervised domain adaptation (UDA) has recently gained attention. UDA methods aim to learn a domain-invariant feature space by bridging the labeled source domain and unlabeled target domain, as shown in Figure 1. UDA methods can be divided into two main categories: (i) domain discrepancy-based methods, where domain-invariant features are found by fine-tuning the model and minimizing domain shift using statistical measures, and (ii) adversarial-based methods using a generative model, where domain-invariant features are learned by encouraging domain confusion using a discriminator network. This method is more complex, as the discriminator needs to be trained from scratch, and hence it takes more training time.

Although deep transfer learning-based UDA approaches have seen a lot of success so far, they still face challenges that need to be overcome to improve their performance. The present study mainly focuses on aligning the marginal or conditional distributions and utilizing the pre-trained model for transferability. Transferability depends on the relatedness and size of the source dataset and target dataset. Transferability plays a significant role in fine-tuning the network to improve the performance of the target task; otherwise, negative transfer or overfitting may occur and degrade performance [4]. In domain adaptation, it is unclear how to efficiently fine-tune the model using the feature transferability across the domains. Moreover, the size of the dataset is also not balanced to improve the marginal probability of the task. Additionally, the present study uses various asymmetric statistical distribution measures [5,6,7,8,9]. Furthermore, the majority of current research in object detection utilizes the de-facto object detection model, Faster R-CNN [10], which is a two-stage network.

In this paper, our goal is to develop a unified unsupervised domain adaptation network for image classification and object detection that can learn more transferable feature representations and effectively reduce the domain shift problem. Firstly, we propose a guided transfer learning (GTL) approach to measure transferability between the source and target domains, called the *τ-score* metric. The *τ-score* is obtained without training the deep network. Instead, we use the deep CNN for feature extraction only to calculate the *τ-score*, saving expensive computation time before the actual training of domain adaptation. Moreover, it is simple to understand and interpret and can be employed in various deep transfer learning CNNs. The *τ-score* has been utilized to identify the layer of the network to fine-tune, instead of selecting only the last few fully connected layers or randomly from the entire network. Secondly, we utilize the weighted cross-entropy loss for domain adaptive image classification and object detection to handle the class imbalance problem. Lastly, we employ the JS (Jensen–Shannon) divergence to calculate the domain discrepancy loss between the domains. The JS-divergence is symmetric and finite, and can be used to calculate the distance between the two distributions. Our domain adaptive object detection method is not dependent on the detection model, such as Faster R-CNN, as it does not consider the regional proposal loss to learn the domain invariant features. Our model only considers the image-level and object-level features to reduce the domain shift in object detection and can be implemented with a two-stage or one-stage object detection network.

The main contributions can be summarized as follows.

- To the best of our knowledge, we propose the first-of-its-kind layer selection strategy using the Guided Transfer Learning approach to fine-tune the domain adaptation network and maximize feature transfer between domains.
- We employ JS-Divergence to reduce the feature distribution gap between source and target domains.
- We introduce the weighted cross entropy loss to tackle the class imbalance problem.
- We further propose a robust object detection UDA framework that is applied to the two-stage Faster R-CNN and single-stage SSD (Single Shot multibox Detector) object detector effectively.
- We conduct extensive experiments on benchmark datasets to validate the performance of our UDA image classification and object detection method, compare it with state-of-the-art (SOTA) methods, and obtain promising results. Moreover, we also demonstrate ablation studies to show the impact of each component in our proposed framework. Furthermore, we present the first-of-its-kind Indian Vehicle dataset for domain adaptive object detection task to evaluate the adaptivity of our object detector in the new domain.

The rest of the paper is organized as follows: Section 2 discusses recent work on UDA-based image classification and object detection methods. The proposed framework for domain adaptive image classification and object detection is explained in Section 3. Section 4 presents the result analysis of both proposed methods with various experiments. Finally, Section 5 summarizes the paper with the conclusion and shows the future directions.

## 2. Related Work

The problem of transferring knowledge from a labeled source domain to an unlabeled target domain is said to be solved by unsupervised domain adaptation. Significant research contributions have been put into supervised and semi-supervised domain adaptation methods. In recent years, increasing research efforts are focused on unsupervised domain adaptation methods that use deep learning architectures to improve the performance of image classifiers and object detectors. To reduce the domain divergence between the source and target domain, there are mainly two main types of UDA methods that have gained significant attention: discrepancy-based UDA methods and adversarial-based UDA methods. In this section, we describe recent works on these approaches for domain adaptive image classification and object detection.

### 2.1. Unsupervised Domain Adaptive Image Classification

#### 2.1.1. Discrepancy-Based Approaches

In discrepancy-based methods, domain adaptation is achieved by minimizing the distance between domain distributions using statistical measures to find domain invariance features.

Ghifary et al. [11] introduced the maximum mean discrepancy (MMD) metric for feedforward neural networks with one hidden layer. The MMD measure reduces the mismatch in the latent space distribution between domain representations. Tzeng et al. [5] employed two AlexNet [12] CNNs in the deep domain confusion network (DDC) for source and target domains with shared weights. An adaptation layer with the MMD metric measures domain difference and optimizes the network for classification loss in the source domain. Long et al. [13] developed the deep adaptation network (DAN) to match marginal distributions across domains by adding adaptation layers and evaluating different kernels. A joint adaptation network (JAN) [14] introduced a joint maximum mean discrepancy (JMMD) and applied it in various domain-specific layers of ResNet-50 [15] to find domain invariance features. Yoo et al. [16] recently presented a weighted MMD model that includes an additional weight for each class in the source domain when the target domain class weights are different. In contrast to MMD, Sun et al. [17] proposed a CORrelation ALignment (CORAL) loss function for deep neural networks, which aligns the second-order statistics across domains and minimizes the domain shift. The Contrastive Adaptation Network (CAN) [7] utilized a new metric contrastive domain discrepancy (CCD), which optimizes the intra- and inter-class discrepancy across the domains and trains the CAN in an end-to-end manner. Lee et al. [18] used the task-specific decision boundary in unsupervised domain adaptation to align feature distributions across domains using sliced Wasserstein discrepancy (SWD). Deng et al. [19] proposed a similarity-guided constraint (SGC) in the form of a triplet loss, which is integrated into the network as an additional objective term to optimize the network. Ref. [20] introduced the balanced weight joint geometrical and statistical alignment (BW-JGSA) for UDA to minimize the distribution divergence between marginal and conditional distributions across domains. In order to discover domain-invariant feature representations, Xie et al. [21] used the Wasserstein distance between the two distributions collaboratively and presented the collaborative alignment framework (CAF) to minimize the global domain discrepancy and retain the local semantic consistency. Wang et al. [22] proposed the manifold dynamic distribution adaptation (MDDA) to learn the domain-invariant transfer classifier in the target domain using the Grassmann manifold.

#### 2.1.2. Adversarial-Based Approaches

Adversarial-based methods train discriminator networks to confuse the domain distributions. The domain-adversarial neural network (DANN) was first introduced in [23] for use in adversarial training by a gradient reversal layer (GRL). DANN uses shared feature extraction layers to reduce label prediction loss and GRL to maximize domain confusion loss. Adversarial discriminative domain adaptation (ADDA) [24] unties the weights and initializes the target model parameters with the pre-trained source model. Learning domain-specific feature extractions makes ADDA more adaptable. ADDA minimizes source and target representation distances by iteratively reducing the generative adversarial network (GAN)-based loss function. Cao et al. presented the selective adversarial network (SAN) [25] to handle transfer learning for small domains by filtering outlier source classes and matching data distributions in the common label space by separating the domain discriminator into several class-wise domain discriminators, which reduces negative transfer and promotes positive transfer. In [26], the feature generator is learned by augmenting the source domain data, and the minimax algorithm is employed to find the domain invariant feature. Wasserstein distance is used to measure domain distance in the discriminator by Shen et al. [9]. and improved the feature extractor network to find the invariant features in an adversarial manner. In [27], a feature extractor generates target features that are similar to the source, while discriminators are trained to increase the discrepancy to recognize target samples outside the source’s support. Zhang et al. [28] introduced Domain-Symmetric Networks (SymNets) for domain adaptation. SymNet was built on the symmetric source and target task classifiers and an extra classifier that shares layer neurons. They proposed a unique adversarial learning method based on a two-level domain confusion method to train the SymNet. The category-level confusion loss tried to reduce the object-level loss by forcing intermediate network features to be invariant. The Hierarchical Gradient Synchronization Domain Adaptation (GSDA) [29] method was presented to align the domain hierarchically including global alignment and local alignment. Local alignment is performed using class-wise alignment. In [30], the authors employed a Hybrid Adversarial Network (HAN) with a classification loss to train the discriminative classifier using adversarial training to find the transferable features across domains. To improve target discrimination, structural regularization deep clustering (SRDC) [31] combines the clustering of features of an intermediate network with structural regularisation and a soft selection of less dissimilar source samples. Na et al. [32] provided a solution by augmenting several intermediate domains using a fixed ratio-based mixup approach to bridge the source and target domains (FixBi). They trained the source-leading and target-leading models that shared common characteristics. Pei et al. [33] introduced a multi-adversarial domain adaptation (MADA) technique to leverage multiple domain discriminators to capture the fine-grained alignment of multimodal structures of the source and target domains. Pinheiro et al. [34] presented an end-to-end similarity learning network (SimNets) method to learn a pairwise similarity function for evaluating the similarity between prototype representations of each class. Long et al. [35] proposed a conditional domain adversarial network (CDAN) that uses multilinear conditioning to capture the cross-covariance between feature representations for discriminability and classifier predictions for classification. Chen et al. [36] introduced the discriminator-free adversarial learning network (DALN), which can use the predicted discriminative information for feature alignment and employs nuclear-norm Wasserstein discrepancy (NWD) for performing discrimination. Table 1 presents a comparative summary of the existing state-of-the-art methods of domain adaptation for image classification.

### 2.2. Unsupervised Domain Adaptive Object Detection

In past decades, CNN-based object detection methods have shown significant improvements applied to various datasets and have been successfully utilized in many computer vision applications. Object detection algorithms are categorized into two-stage [10,41,42] and one-stage [43,44,45] object detectors. These object detection algorithms require the annotated datasets and obtain marginal reductions in performance when applied to another domain with the same label space. Recently, research efforts have been focused on aligning domains for object detection tasks.

Chen et al. [46] proposed the first-of-its-kind domain-adaptive object detection algorithm using Faster R-CNN with adversarial feature adaptation to minimize distribution divergence at the image and instance levels. Saito et al. [47] employed strong local and weak global alignments to propose strong-weak distribution alignment (SWDA) for shallow receptive fields and image-level features on deep convolutional layers respectively.

Zhu et al. [48] aligned the region proposal generated by the Faster R-CNN detectors from the source and target domain by applying the k-means clustering algorithm using selective cross-domain alignment (SCDA). Zheng et al. [49] performed adversarial feature learning with the coarse-to-fine adaptation (CFA) approach by proposing the attention-based region transfer (ART) and prototype-based semantic alignment (PSA) to learn domain invariant features. In [50], the authors applied image-level alignment at multiple layers of the backbone network and trained it using an adversarial manner with the multi-adversarial Faster R-CNN (MAF) framework. Kim et al. [51] trained the domain adaptive object detector by augmenting the samples from both domains and learned the domain invariant features across the domains. Conditional Domain Normalization (CDN) is introduced to reduce the domain divergence between the domains in [52]. CDN encodes characteristics from different domains into a latent space with the same domain attribute. It is applied in multiple convolutional layers of the detection model to align the domains. A Hierarchical Transferability Calibration Network (HTCN) is employed by Chen et al. [53] to learn the transferability and discriminability of feature representations hierarchically. They proposed three components consisting of Weighted Adversarial Training, Context-aware Instance-Level Alignment, and local feature masks. Rodriguez et al. [54] proposed domain adaptive object detection using the style consistency (ODSC) framework based on SSD [43] and trained the framework with the style transfer method for pixel-level adaptation and pseudo labeling to reduce the negative samples from the unlabeled target domain. Wang et al. [55] introduced the sequence feature alignment (SFA) technique on the deformable detection transformer (DefDETR) network [45] to adapt the domain discriminative features. The SFA comprises two distinct modules: a token-wise feature alignment (TDA) module and a domain query-based feature alignment (DQFA) module. Zhou et al. [56] utilized the multi-granularity alignment (MGA) with three-level domain alignment losses to learn the domain-invariant features between the domains including pixel-level, instance-level, and category-level. The MGA method has been developed based on faster R-CNN and fully convolutional one-stage (FCOS) [44] backbone detectors. Gong et al. [57] introduced the O^2^net method with the object-aware alignment (OAA) and optimal transport-based alignment (OTA) modules to apply pixel and instance levels domain alignment loss. Table 2 summarizes the existing state-of-the-art methods for domain adaptation in object detection.

## 3. Methodology

This section presents the proposed algorithm (Domain Adaptation using Guided Transfer Learning—DAGTL) in detail. First, the notations are defined for domain adaptation, and the problem statement is formulated. Then, we introduce the guided transfer learning approach to select the layer from which the model is fine-tuned. Next, we explain two proposed approaches: Domain Adaptation using Guided Transfer Learning for image classification (DAGTL-IC) and Domain Adaptation using Guided Transfer Learning for object detection (DAGTL-OD). Finally, we present the overall objective functions to minimize the loss for domain adaptive image classification and object detection.

### 3.1. Problem Formulation

Let *x* denote the input image and *y* denote the corresponding label of the image. We define the domain as *D = {X, P(x)}*, where *X* is the feature space *X = {x_1_, x_2_, …, x_n_}* and *P(x)* is a marginal probability distribution. The proposed algorithm is designed to address the problem of unsupervised domain adaptation and aims to adapt the features from a label-rich source domain $D^{s}=\left\{X^{s},P^{s}\left(X^{s}\right)\right\}$ to a label-scarce target domain $D^{t}=\left\{X^{t},P^{t}\left(X^{t}\right)\right\}$. The source domain has adequate labeled samples, which are denoted as $D^{s}=$ ${\left\{\left(x_{n}^{s},y_{n}^{s}\right)\right\}}_{n=1}^{N^{s}}$ where $x^{s}$ is source sample, $y^{s}$ is the associated label of the given source sample and $N^{s}$ is the number of samples available in the source domain. Furthermore, the target domain contains unlabeled samples, which are denoted as $D^{t}=$ ${\left\{\left(x_{n}^{t}\right)\right\}}_{n=1}^{N^{t}}$, where $N^{t}\ll N^{s}$. Usually, the source and target data distribution spaces are different in domain adaptation. However, the label space of samples is the same in both domains, the label space $\Upsilon =\left\{1,\;2,\;\ldots ,C\right\},\;\left|\Upsilon \right|=$ number of labels. The task is also identical. The goal of our work is to learn domain invariant features by aligning the source and target domain features in a common latent space and to minimize the domain discrepancy between the domains. Thus, the target task performance increases with unlabeled target data.

### 3.2. Guided Transfer Learning Approach

The deep domain adaptation utilizes the deep convolutional neural network to improve the performance of the target task for image classification and object detection. Our proposed work is based on the discrepancy-based deep domain adaptation approach. In this approach, transfer learning is performed by fine-tuning the convolutional neural network using target data to minimize the distribution shift between the source and target domains.

The convolutional neural network requires a large amount of labeled data to train the model from scratch. However, this assumption does not necessarily hold in real life due to the scarcity of annotated data. Additionally, deep learning algorithms are data-dependent for a particular domain or problem. These algorithms require re-training when the domain shifts. The transfer learning approach utilizes the knowledge from the source domain to improve the performance of the target task in the related target domain. Therefore, transfer learning substantially enhances the model performance and decreases the model development time. However, the effectiveness of transfer learning depends on the size of the target domain and the similarity between domains. In domain adaptation, source, and target domains share common label space but feature distributions are different and the size of target data is also small. Therefore, we applied the fine-tuning strategy of transfer learning on the target domain using deep convolutional neural networks. The convolutional neural networks are designed with a hierarchical representation of layers, including convolutional, pooling, and fully connected layers. The initial layers of CNNs learn simple features of an object, while higher-level layers attempt to learn more complex and abstract features. In the fine-tuning strategy of transfer learning, initial layers are frozen to reuse low-level features, and deeper layers are fine-tuned to train the parameters based on the relatedness of the source and target domain. The transfer learning algorithm trains partial layers of the pre-trained network. Selecting partial layers of pre-trained CNN models depends on the correlation between the target and source domain. Therefore, it is essential to investigate the effective layer selection strategy from which layer to freeze and fine-tune the model to perform the target task efficiently, rather than selecting layers empirically or randomly.

**Figure 2:**
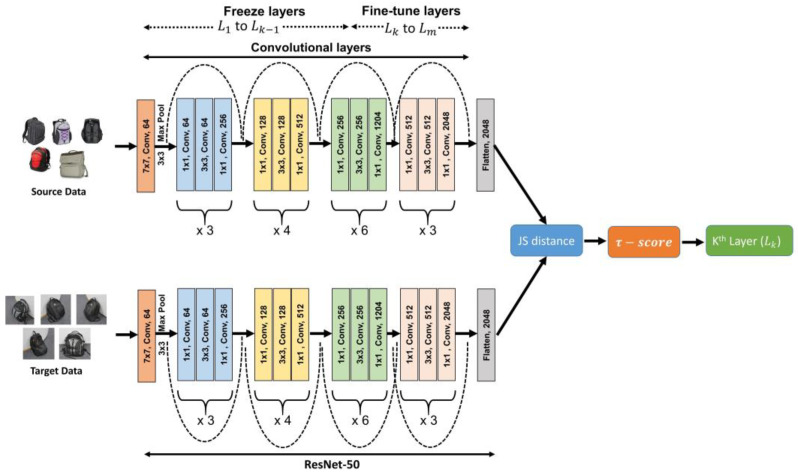
Guided Transfer Learning approach for layer section from where to freeze and fine-tune the model.

The proposed guided transfer learning approach is illustrated in Figure 2 and it is based on the ResNet-50 (Residual Network) architecture. This approach calculates the transferability score (τ−score) to determine the optimal layer for fine-tuning the proposed algorithms and to guide the network on which layer to freeze and fine-tune during the training process. The transferability score (τ−score) measures the effectiveness of the transfer learning algorithm to transfer the knowledge from the source domain $D^{s}$ to the target domain $D^{t}$ which can improve the performance of the target task $T^{t}$. The τ−score indicates the similarity between source and target domains, which is used to identify the layers of the CNN to be frozen or fine-tuned. The τ−score value ranges in [0, 1]. Intuitively, if τ−score is near to 1, it shows less similarity between the $D^{s}$ and $D^{t}$ due to the high distribution distance between the domains. Thus, few or none of the CNN layers’ parameters can be transferred from the source model to the target model. If τ−score is near to 0, it indicates high similarity between the $D^{s}$ and $D^{t}$ and the CNN layers’ parameters can be transferred from the source model to the target model.

Let L define the layers of the CNN where $L=\left\{L_{1},L_{2},\;\ldots ,L_{k},\ldots L_{m}\right\}$, m indicates the total number of layers which includes convolutional and fully connected layers. The key idea of this approach is to find the *k*th layer ($L_{k}$) of the CNN from which, layers $L_{1}$ to $L_{k-1}$ are frozen and layers $L_{k}$ to $L_{m}$ are fine-tuned. The layer ($L_{k}$) is identified using Algorithm 1.

**Algorithm 1:** Guided Transfer Learning (GTL) approach to find the *k*th layer
**Input:** labeled source domain $\left(X^{s},\;Y^{s}\right)$, unlabeled target domain data $\left(X^{t}\right)$
**Output:** *k*th layer ($L_{k}$)1:**Take five random samples (**$R_{s}$**) from the Source domain** $(D^{s}$**) and Target domain** $(D^{s}$**) for each label** $\left(\Upsilon \right).$ Here, each label is considered as per the labeled data available in the source domain. Since label space is the same in both domains, five random samples have been selected from the target domain. Here, we consider whole images for the image classification problem and cropped images as per the bounding box for the object detection problem.2:**Pass all samples through the ResNet-50 network up to the last convolution layer to generate a flattened feature vector and apply the mean on five feature vectors of the same labeled images.** $F_{ij\;}^{s}$—source feature vectors, $F_{ij\;}^{t}$—target feature vectors, where *j* = 1 to 5 (#samples), *i* = 1 to $\left|\Upsilon \right|$ (#labels). The mean of five samples’ feature vectors of each label is calculated using Equations (1) and (2). (1)$$F_{i\;}^{s}=\sum_{j=1}^{R_{s}}F_{ij\;}^{s}/R_{s}\;$$
(2)$$F_{i\;}^{t}=\sum_{j=1}^{R_{s}}F_{ij\;}^{t}/R_{s}$$3:**Apply JS (Jensen–Shannon) distance among** $F_{i\;}^{s}$** source feature vectors and **$F_{i\;}^{t}$** the target feature vector of each label to find the similarity score **$\left(SC_{i}\right)$** between each source label to the target label.** Transferability measures should be symmetric in the domain adaptation because the label space is common in both domains and the distance between two feature vectors is computed using JS-Divergence. (3)$$SC_{i}=\sqrt{JS(F_{i\;}^{s}\;||\;F_{i\;}^{t})}$$where JS-Divergence is the measure of the difference between two probability distributions. In particular, it assesses the similarity between two probability distributions by calculating the average Kullback–Leibler (KL) Divergence between each distribution. The JS-Divergence can be calculated as follows between the two distributions P and Q: JS(P || Q) = 1/2 × KL(P || M) + 1/2 × KL(Q || M)(4)KL(P, M) is the KL-Divergence between the two probability distributions P and M. Similarly, KL(Q, M) is the KL-Divergence between the two probability distributions P and M; where M is the average of P and Q and is defined as M = 1/2 × (P + Q)(5)4:**Calculate Transferability Score (**τ−score**).** τ−score is computed by taking an average of the similarity score of each label, calculated in step-3. (6)$$\tau -score=\overset{\left|\Upsilon \right|}{\underset{i=1}{\sum }}SC_{i}/\left|\Upsilon \right|$$5:**Find the *k*th layer.** Layers $L_{1}$ to $L_{k-1}$ are frozen and layers $L_{k}$ to $L_{m}$ are fine-tuned during the training process. (7)k=⌊ τ−score×m ⌋

### 3.3. Proposed Approach

In this paper, we introduce the discrepancy-based unsupervised domain adaptation framework using GTL for image classification (DAGTL-IC) and object detection (DAGTL-OD). In unsupervised domain adaptation, the target domain cannot be directly used to fine-tune the model for the target task due to unlabeled data. In the proposed approach, we utilize labeled source data and unlabeled target data with the same categories of class. The objective is to reduce the domain distribution distance using domain loss functions and to learn better transferable feature representations, which can improve the overall performance of the target task. This is achieved by fine-tuning the ResNet-50 network for image classification and fine-tuning the Faster R-CNN and SSD for object detection. The ResNet-50 network is used as the backbone to extract features in object detection models. The ResNet-50 model comprises five stages, each stage contains a convolution and Identity block. Each convolution block and identity block have three convolution layers. There are forty-nine convolutional layers and one fully connected layer. A detailed explanation of the proposed approaches is given in subsequent sections.

#### 3.3.1. Unsupervised Domain Adaptation for Image Classification (DAGTL-IC)

**Figure 3:**
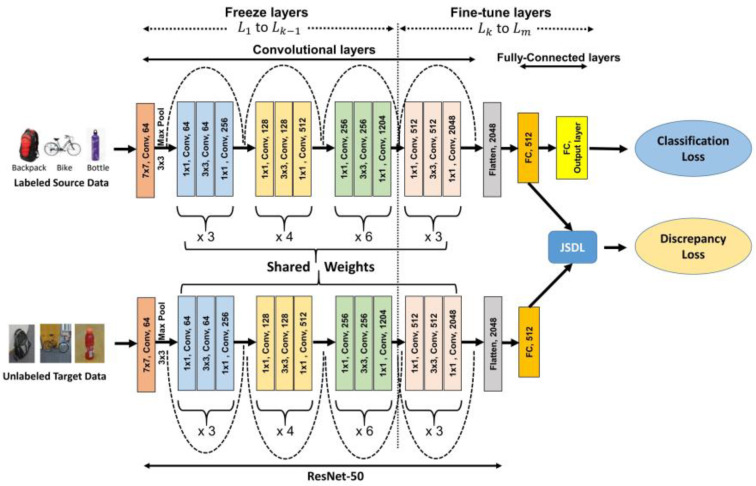
Proposed architecture for domain adaptive image classification using the guided transfer learning approach.

The proposed DAGTL-IC architecture consists of two streams of ResNet-50 networks for the source and target domains with weight parameter sharing, as illustrated in Figure 3. We pass labeled source domain data to the source network and unlabeled target domain data to the target network. The last layer of the ResNet-50 network, which is a fully connected layer with 1000 neurons, is omitted. The last convolutional layer is converted to a flattened layer and comprises 2048 neurons. After this flattened layer, we added one additional bottleneck layer with 512 neurons. The bottleneck layer takes the input from the flattened layer of both the source and target streams and computes the domain discrepancy loss using JS-Distance which reduces the distance between the domain distributions and finds domain invariant features. The last layer of ResNet-50 is modified based on the number of classes available in the domains for classification. The proposed architecture is trained using the guided transfer learning approach based on the layer selection strategy to select which layers are kept frozen and which layers are fine-tuned as mentioned in Section 3.2, instead of a random selection of layers. The first stream uses labeled source data to train a classifier using the cross-entropy loss function and both streams use data from both domains to find the discrepancy loss using JS-Distance between the domains. These loss functions are explained in the following section and Algorithm 2 presents the steps to perform the proposed approach DAGTL-IC. During inference, the fine-tuned network is directly applied to the target domain for the target task.

#### 3.3.2. Loss Functions for Domain Adaptive Image Classification

1. Classification loss
  Classification loss is calculated using the first stream of the architecture. It uses the labeled data from the source domain only as the target domain does not have labeled data. The features extracted from the last flattened layers are fed into the output layer with a softmax classifier to optimize the classification loss. The classification loss function can be written as follows:
  (8) $$L_{c}=-\frac{1}{N^{s}}\overset{n}{\underset{i=1}{\sum }}w_{i}\times \left(y_{i}\log\left(\hat{y}\right)\right)$$
  where $L_{c}$ is the classification loss in the source domain which represents the cross-entropy loss function, $y_{i}$ is the actual label, $\hat{y}\;$ is the predicted label, and $w_{i}$ is the weight parameter used to handle the class imbalance problem.
  The classifier is expected to train well the conditional probability of input data $X_{i}^{s}$ to $Y_{i}^{s}$ in the source label space. However, this assumption holds true only when the labeled data are equally divided among the number of classes. In the dataset of domain adaptation, it is observed that the data are not equally divided, resulting in a biased classification. In order to mitigate this situation, we introduced the weight $w_{i}$ to each of the classes to improve the performance of the classifier. $w_{i}$ can be defined as follows for each category:
  Let $F=\left\{f_{c_{1}},f_{c_{2}},\ldots ,f_{c_{\left|\Upsilon \right|}}\right\}$, this represents the frequency of each category.
  (9) $$w_{i}=\frac{f_{max}}{f_{c_{i}}}$$
  where $i=1\;to\;\left|\Upsilon \right|;\;f_{max}=max\left(F\right)$.
  - F is a set of frequencies (number of samples) of each category, where $f_{c_{i}}$ represents the frequency of the *i*th category.
  - $\left|\Upsilon \right|$ is the total number of categories in the dataset.
  - $f_{max}$ is the maximum frequency in *F*, i.e., the highest number of samples among all categories.
  - $w_{i}$ is the weight for the *i*th category, which represents how important that category is in the dataset.
  - The formula calculates $w_{i}$ by taking the ratio of $f_{max}$ and $f_{c_{i}}$.
  The intuition behind this equation is to assign lower weights to categories that appear more frequently, and higher weights to categories that occur less frequently. This equation balances the importance of each category during training and reduces the class bias problem due to an imbalanced dataset.
2. Dyomain discrepancy loss
  The domain discrepancy loss is computed between the bottleneck layers of both domain streams. To minimize the distance between the domains, JS-Distance is employed to learn the domain invariant features. We intend to transfer as much knowledge as possible from the source domain to the target domain by minimizing the domain alignment loss. The feature vectors of the bottleneck layers of the source and target domains are denoted as $X_{b}^{s}$ and $X_{b}^{t}$ respectively. This loss function can be calculated as follows:
  (10) $$L_{d}=\sqrt{JS(X_{b}^{s}\;||\;X_{b}^{t})}$$
  JS-Distance is the square root of the JS-Divergence and its value ranges between 0 (highly similar distributions) and 1 (maximally different distributions) when using a base-2 logarithm. JS-Divergence is a method used to measure the similarity between two probability distributions. The reasons to use JS-Divergence are two folds. These are: (i) It is a symmetric version of KL-Divergence and can be used to calculate the distance between distributions because it has a finite range between 0 and 1. (ii) It is a kind of average between two distributions, thus two distributions are equally participating to find the domain invariant features.
3. Overall objective loss function for domain adaptive image classification
  To achieve efficient domain adaptation in image classification, the aim is to minimize the distance between the domains and train a classifier that can be transferred across the domains. To meet both these criteria, an integrated approach is used by combining the classification loss and domain shift loss as an overall objective loss function with a trade-off parameter. The objective is to minimize the overall loss. After reducing the overall loss to a minimum, the trained model is directly applied to the target domain. The overall objective loss function for image classification is given as follows:
  The overall objective loss function of DAGTL-IC:
  (11) $$min(L_{DAGTL-IC}=L_{c}+\lambda L_{d})$$
  $L_{c}$ denotes the classification loss in the source domain, $L_{d}$ represents the domain discrepancy loss between the domains and *λ* is the trade-off parameter; λ > 0.

**Algorithm 2:** Unsupervised Domain Adaptation for Image Classification (DAGTL-IC)
**Input:** labeled Source domain $\left(X^{s},\;Y^{s}\right)$, Unlabeled target domain data $\left(X^{t}\right)$, regularization parameters *λ*. **Output:** Domain invariant features F, Classifier *C*.1:Configure the CNN. Initialize the ResNet-50 model until the last convolutional layer, then add a bottleneck layer of 512 neurons. Finally, add an output (classification) layer with a number of neurons that is equal to the number of categories in the dataset.2:Find the *k*th layer $\left(L_{k}\right)$ using a guided transfer learning approach, according to Algorithm 1.3:Freeze the layers $L_{1}$$\;\mathrm{to}\;L_{k-1}$ and fine-tune the layers $L_{k}$$\;\mathrm{to}\;L_{m}$ during the training process.4:
**Repeat**
5:Sample mini-batch from the source domain with labeled data and the target domain with unlabeled data6:Feed the sampled mini-batch and calculate domain discrepancy loss $\left(L_{d}\right)$, classification loss $\left(L_{c}\right)$ and the overall objective loss function $L_{DAGTL-IC}.$
7:Update the parameters of the network by minimizing the overall loss $L_{DAGTL-IC}$ using the stochastic gradient descent (SGD) method.8:**Until** $L_{DAGTL-IC}$ converges.9:Return trained Classifier C.


#### 3.3.3. Unsupervised Domain Adaptation for Object Detection (DAGTL-OD)

**Figure 4:**
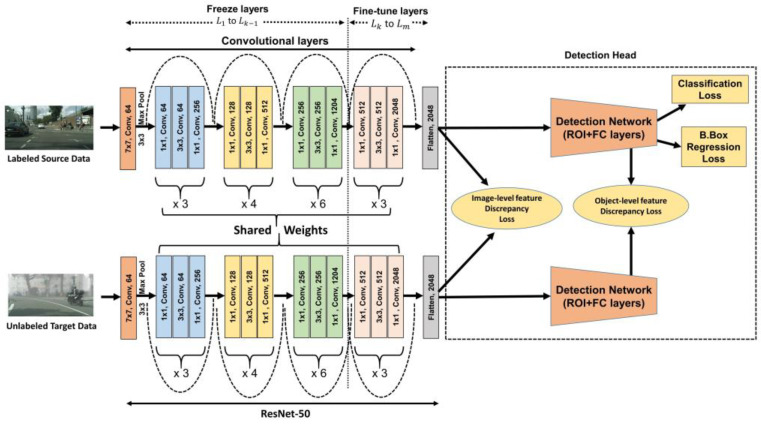
Proposed architecture for domain adaptive object detection using the guided transfer learning approach.

The proposed DAGTL-OD architecture consists of two streams of the object detection network for source and target domains as illustrated in Figure 4. Faster R-CNN and SSD object detection networks are utilized in the proposed method. Faster R-CNN is a two-stage object detection network and has three main components: backbone CNN layers, a Region Proposal Network (RPN) for generating region proposals, and a Region-of-Interest (ROI) based classifier Network (RCN) for the classification of objects and predicting bounding boxes. SSD is a one-stage object detection network that directly classifies objects from the features map. Our approach can be applied to both types of object detection networks. We pass the annotated images of the source domain and unlabeled images of the target domain to the proposed model. We use the ResNet-50 model as the backbone of the detection network and its features are shared between both streams. Instead of selecting random layers to fine-tune, we utilize the guided transfer learning strategy mentioned in Section 3.2 to train the base layers of the network. The feature vectors from the flattened layers of ResNet-50 are then passed to the detection head to predict the object coordinates and their categories. Both networks are jointly trained to minimize the loss function $\left(L_{det\_net}\right)$, which is composed of the classification loss $\left(L_{cls}\right)$ and regression loss $\left(L_{reg}\right)$ for object detection in the source network. The regression loss is used to find the accurate bounding box of the objects in the given image. Moreover, we utilize two types of domain losses in the proposed network training: image-level domain discrepancy loss and object-level domain discrepancy loss. These losses and the overall objective function for domain adaptive object detection are presented in the following section and Algorithm 3 presents the steps to perform the proposed approach DAGTL-OD. Once the domain invariant features are learned from the training of both networks, these features are directly utilized to detect objects in the target domain by the fine-tuned network.

#### 3.3.4. Loss Functions for Domain Adaptive Object Detection

1. Detection loss
  The object detection model is trained with classification loss and regression loss. Classification loss $\left(L_{cls}\right)$ and regression loss $\left(L_{reg}\right)$ are used to classify the object with a label and bounding box for better object localization from the ROIs. Classification loss is calculated as per Equation (8) with weight to handle the class imbalance problem. Regression loss is computed by applying the smooth L1 loss function to the difference between the predicted and ground truth bounding box values. These losses are computed in the source network, as this network is trained with labeled data only. The loss function of the detection model is written as follows:
  (12) $$L_{det\_net}=L_{cls}+L_{reg}$$
2. Domain discrepancy loss
  In object detection, there are two important aspects for reducing the shift between the domains: whole image differences like scale, illumination, etc., and particular objects of the image differences like scale, and appearance. To align the distribution between domains, we introduce the two types of losses in the proposed network training: image-level domain discrepancy loss $L_{img}$ and object-level domain discrepancy loss $L_{obj}$. The image level discrepancy loss is calculated using JS-Divergence between the features extracted from the flattened layer of the source and target networks. This loss eliminates the distance between the distribution of both domains at the image level and learns the domain invariant features across the domains. Let $X_{f}^{s}$ and $X_{f}^{t}$ denote the feature vectors of the flattened layer from the source and target networks respectively. The image-level domain discrepancy loss can be written as
  (13) $$L_{img}=\sqrt{JS(X_{f}^{s}\;||\;X_{f}^{t})}$$
  The object-level features are obtained from the vectors of the region of interest. These feature vectors from both domains are utilized to compute $L_{obj}$ using the JS-divergence. However, there is not a fixed number of ROI vectors in both domains. Thus, the object-level domain discrepancy loss can be written for the *j*th ROI vector in the *i*th image as follows.
  (14) $$L_{obj}=\frac{1}{N_{roi}}\underset{i,j}{\sum }\sqrt{JS(X_{roi_{i,j}}^{s}\;||\;X_{roi_{i,j}}^{t})}$$
3. Overall objective loss function for domain adaptive object detection
  To obtain an effective domain adaptive object detector, we attempt to reduce the domain shift gap across the domains including the classification and regression loss of the object detection model. We combine the detection loss and domain shift loss as an overall objective loss function with a trade-off parameter, and our goal is to minimize the total loss. After reducing the overall loss to a minimum, the trained detector model is directly applied to the target domain. The overall objective loss function for object detection is as follows.
  The overall objective loss function of DAGTL-OD:
  (15) $$min(L_{DAGTL-OD}=L_{det\_net}+\lambda (L_{img}+L_{obj}))$$
  $L_{det\_net}$ defines the object detection loss, which includes classification loss and regression loss, $L_{img}$ denotes the image-level domain discrepancy loss, $L_{obj}$ represents the object-level domain discrepancy loss, and λ is the trade-off parameter; λ > 0.

**Algorithm 3:** Unsupervised Domain Adaptation for object detection (DAGTL-OD)**Input:** labeled Source domain $\left(X^{s},\;Y^{s}\right)$, Unlabeled target domain data $\left(X^{t}\right)$, regularization parameters *λ*. **Output:** Domain invariant features at image-level $\left(F_{img}\right)$ and object-level $\left(F_{obj}\right)$, Detector D.1:Configure the object detection model. Initialize the backbone network as a ResNet-50 model until the last convolutional layer. Add detection head (Faster R-CNN/SSD).2:Find the *k*th layer $\left(L_{k}\right)$ from the ResNet-50 network using the guided transfer learning approach according to Algorithm 1.3:$\mathrm{Freeze}\;\mathrm{the}\;\mathrm{layers}\;L_{1}$$\;\mathrm{to}\;L_{k-1}$ and fine-tune the layers $L_{k}$$\;\mathrm{to}\;L_{m}$ of ResNet-50. including the whole detection head during the training process.4:
**Repeat**
5:Sample mini-batch from the source domain with labeled data and the target domain with unlabeled data6:Feed the sampled mini-batch and calculate object detection loss $\left(L_{det\_net}\right)$, image-level domain discrepancy loss ($L_{img}$), object-level domain discrepancy loss ($L_{obj}$) and overall objective loss function $L_{DAGTL-OD}$.7:Update the parameters of the network by minimizing the overall loss $L_{DAGTL-OD}$ using the SGD method.8:**Until** $L_{DAGTL-OD}$ converges.9:Return trained detection network D.


## 4. Experimental Analysis

In this section, the proposed DAGTL-IC and DAGTL-OD have been extensively evaluated on four benchmark domain adaptation datasets. Firstly, dataset description and implementation setup along with hyper-parameter value are described. Secondly, the DAGTL approaches are compared with the state-of-the-art domain adaptation methods to examine their performance. Lastly, we analyze the performance by taking various trade-off parameter values and present an ablation study to know the impact of each component on the overall performance of the proposed DAGTL algorithms.

### 4.1. Dataset Description

#### 4.1.1. Office-31

**Figure 5:**
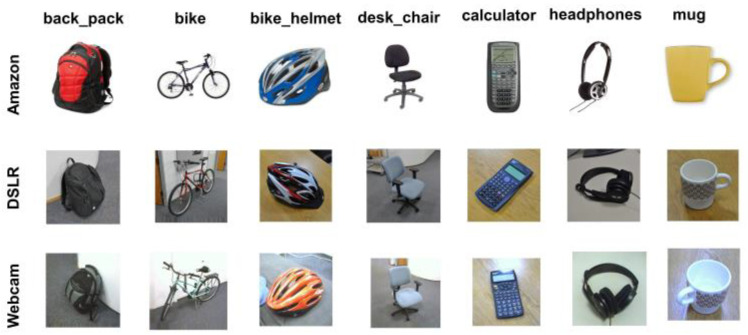
Example images of Office-31 dataset.

Office-31 [37] is a widely used image dataset in domain adaptation for an image classification task. It comprises three distinct domains, namely Amazon (A), Webcam (W), and DSLR (D) with 31 categories and 4110 images in total. Amazon contains 2817 images collected from the amazon.com website with a white background, Webcam has 795 low-resolution images and DSLR covers 498 high-resolution images. This dataset constructs six transfer tasks with one domain as the source and another as the target domain: A → W, A → D, W → A, W → D, D → A, D → W. Sample images are illustrated in Figure 5.

#### 4.1.2. Office-Home

**Figure 6:**
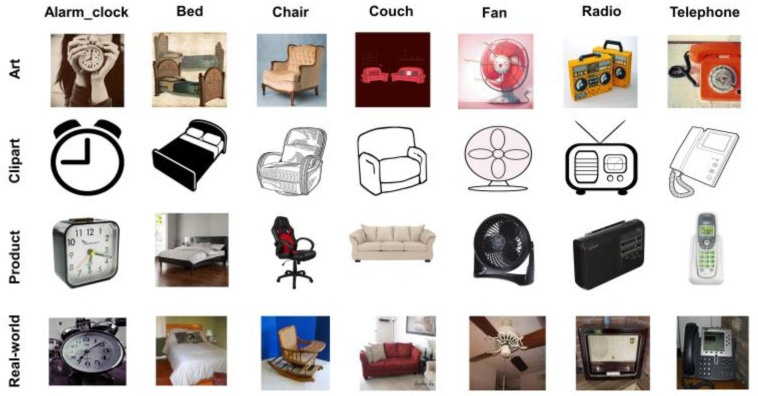
Example images of Office-Home dataset.

Office-Home [38] is a standard benchmark and challenging dataset in visual domain adaptation. It consists of 65 object categories, 15,588 total images, and four domains, namely Art (Ar), Clipart (Cl), Product (Pr), and Real-World (Rw). This dataset contains 2427 artistic images including paintings and sketches, 4365 clip-art images, 4439 product images downloaded from e-commerce websites, and 4357 real-world images taken from cameras having complex backgrounds. Twelve transfer tasks are conducted by selecting one domain as the source and another as the target domain. Sample images are shown in Figure 6.

#### 4.1.3. Cityscapes

**Figure 7:**
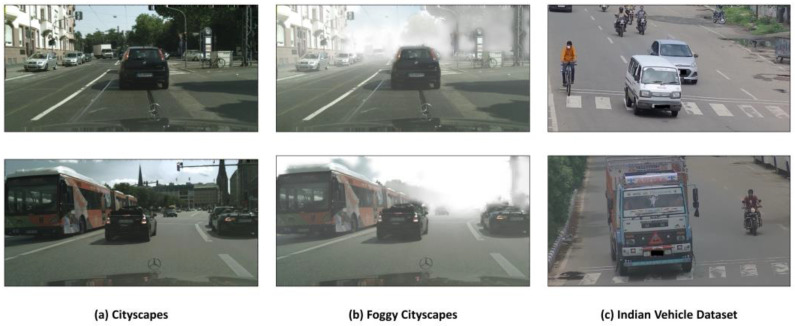
Sample images of object detection dataset—(**a**) Cityscapes; (**b**) Foggy Cityscapes; and (**c**) Indian Vehicle dataset.

Cityscapes [58] is a dataset of real urban street scenes. This dataset is captured through a car dashboard-mounted camera from the urban roads of 50 cities and collects the various object categories, including person, rider, motorcycle (bike), car, bus, truck, train, and bicycle. It contains 3475 images with annotations. We use the standard ratio of training and testing sets as utilized by other researchers to compare our work which is 2975 images as the training set and 500 images as the testing set. Example images are presented in Figure 7a.

#### 4.1.4. Foggy Cityscapes

Foggy Cityscapes [58] is a synthetic dataset generated from the Cityscapes dataset. Three different intensity levels of synthetic fog have been added to analyze the effect of domain adaptive algorithms in adverse weather. It contains the same eight categories of objects with annotations from the Cityscapes dataset. The training set and testing set sizes are also the same, which are 2975 images for training and 500 images for testing. Samples are depicted in Figure 7b.

#### 4.1.5. Indian Vehicle Dataset

The Indian Vehicle dataset is collected from the CCTV cameras of six different crossroads in Vadodara city, Gujarat, India. This dataset contains 3500 images of real road scenes with five categories of objects. This dataset is divided into 2975 images for the training set and 525 images for the testing set. Object categories are car, truck, bus, motorcycle (bike), and bicycle with rectangle annotations. Each image is a color image, and the resolution is 1359 × 720. This dataset is utilized to show the effect of adapting to a new dataset. Samples are illustrated in Figure 7c.

### 4.2. Implementation Details

The proposed DAGTL methods are implemented using the PyTorch deep learning library. The hardware configuration of the system was 64 GB RAM, Nvidia RTX A4000 (16 GB) graphics card, and an Intel Xeon processor. The ResNet-50 architecture has been selected as the backbone network for the proposed DAGTL approaches for two reasons. Firstly, ResNet-50 has a relatively smaller number of parameters compared to other deeper convolutional neural network (CNN) architectures, which makes it computationally efficient, less prone to overfitting, and easier to fine-tune for domain adaptation tasks. Secondly, other existing methods have also utilized ResNet-50 as the backbone network. Therefore, employing ResNet-50 enables a fair comparison of the proposed DAGTL approaches with other state-of-the-art methods. The proposed model is fine-tuned to update the weights from $L_{k}$ convolutional layer to the last layer while keeping the earlier layers from the first convolutional layer to the $L_{k-1}$ convolutional layer frozen as per Table 3. This layer selection strategy is implemented using the guided transfer learning approach mentioned in Section 3.2.

We follow the standard evaluation protocol for our UDA transfer tasks, which is considered in [5,13,23] to implement UDA algorithms. We utilize all the labeled source samples and unlabeled target samples. A mini-batch stochastic gradient descent (SGD) optimizer is used to train the network for 100 epochs with a batch size of 128, momentum of 0.9, and weight decay set to 5 × 10^–4^. The base learning rate is 0.0001, which is used in frozen layers, and the learning rate is set to be 10 times higher than the base learning rate for the layers trained from scratch. Five random experiments are conducted for all the transfer tasks of Office-31 and Office-Home datasets, and the average result in accuracy is reported. A trade-off parameter (*λ*) of 0.8 was chosen from {0.1, 0.2, 0.4, 0.5, 0.6, 0.8, 1, 1.5, 2}, which balances the classification loss and discriminative losses to achieve minimum objective loss value.

We utilize the Faster R-CNN and SSD models as object detection networks with ResNet-50 as the backbone network. We consider a 0.5 IoU threshold for experiments to evaluate mean average precisions (mAP). Training and testing set images are resized to 600 pixels in length. The batch size is set to 16, and the learning rate is 0.001 for 60 epochs and 0.0001 for the remaining 40 epochs. We use stochastic gradient descent (SGD) to train the model with a momentum of 0.9 and a weight decay of 0.0005. We set *λ* = 0.2 and 0.4 for the Cityscapes → Foggy-Cityscapes (C → F) and Cityscapes → Indian Vehicle Dataset (C → I) domain transfer tasks, respectively. The foggy dataset has three variants of fog intensity levels, and we choose the highest intensity level of fog (β = 0.02) as per the baseline algorithms for fair comparison for the C → F domain transfer task.

### 4.3. Results and Discussion

We evaluate the effectiveness of our proposed DAGTL methods by comparing them with various state-of-the-art deep domain adaptation algorithms, which are discussed in the related work and presented in Table 1 and Table 2. We either utilize results reported by other authors in their publications or perform the experiments using publicly provided source codes with the same settings and protocols. The experiments of DAGTL-IC are implemented using two benchmark datasets, namely Office-31 and Office-Home. Furthermore, the experiments of DAGTL-OD are conducted using three benchmark datasets, namely the Cityscapes, Foggy Cityscapes, and Indian Vehicle datasets. The results of DAGTL-IC and DAGTL-OD are discussed in the following sections.

#### 4.3.1. Office-31

Table 4 shows a comparative analysis of the results of DAGTL-IC and previous state-of-the-art deep domain adaptation algorithms on the Office-31 dataset. It can be seen that the average accuracy of our method outperforms all other algorithms. Our method achieves 93.2% average accuracy, which shows a performance improvement of 1.8% compared to the recent FixBi unsupervised domain adaptation algorithm and a significant performance improvement compared to baseline algorithms. There are six transfer tasks in the Office-31 dataset. The result reveals that among these six transfer tasks, our method shows significant improvement in four hard transfer tasks i.e., A → W, W → A, A → D, and D → A, and achieves 97.1%, 82.7%, 97.2%, and 82.9% accuracy, respectively. In these transfer tasks, the similarity of the source domain and target domain is less and there is a large difference in the size of the data between the source domain and the target domain.

#### 4.3.2. Office-Home

Table 5 illustrates the performance analysis of the Office-Home dataset among twelve transfer tasks. It can be seen that the average accuracy of our approach DAGTL-IC is superior to other mentioned algorithms. DAGTL-IC achieves 75.3% average accuracy and outperforms FixBi by 2.6% and baseline algorithms by a significant margin. DAGTL-IC shows substantial improvement in eleven transfer tasks and achieves the highest accuracy. Furthermore, DAGTL-IC achieves the second-highest accuracy in the Rw → Ar transfer task. Additionally, results show that DAGTL-IC outperforms in the transfer tasks where Cl (Clipart) is either the source domain or target domain because Cl (Clipart) has less similarity compared to other domains in this dataset. 

#### 4.3.3. Cityscape → Foggy Cityscapes

**Figure 8:**
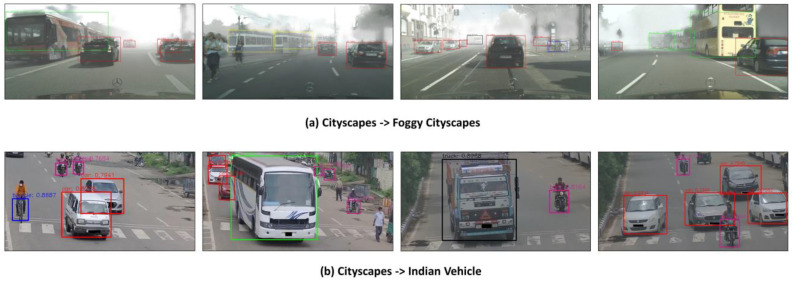
Output images of the proposed DAGTL-OD method using the Cityscapes dataset and adapted on (**a**) Foggy Cityscapes and (**b**) Indian Vehicle dataset.

Weather is a significant cause of domain discrepancy, as changing weather conditions result in a visibly distinct scene. We utilize the Cityscapes and Foggy Cityscapes datasets as the source and target domains, respectively, to assess the model adaptability from normal to foggy weather conditions. Table 6 presents the results of domain adaptive object detection algorithms and their comparison with our DAGTL-OD model. Table 6 reveals that the mean average precision (mAP) of our object detection model outperforms the other baselines. Our model achieves 49.7% mAP when Faster R-CNN is the base detector model, which shows a significant gain of 22.1% compared to DA-Faster and outperforms the state-of-the-art O^2^net [57] by 2.9%. Moreover, our algorithm does not depend on regional proposals to learn domain invariant features through the region proposal loss and depends only on image level and object level features. Thus, we also employ a one-stage object detector SSD to examine the performance of our object detector and achieve promising results among all other results with 51.1% mAP, showing a 4.3% improvement in mAP compared to the state-of-the-art O^2^net. It is worth noting that the performance of our model in each category is highest except for the rider when Faster R-CNN is the base model. This indicates that the DAGTL-OD approach can decrease the domain gap across various objects. Detection samples are shown in Figure 8a. 

#### 4.3.4. Cityscape → Indian Vehicle Dataset

Domain adaptation is essential when changes occur in intra-class variations, camera sensors, geographic areas, or environmental setups. We investigate the adaptability of our method for such changes by considering the Cityscapes dataset as the source domain and the Indian Vehicles dataset as the target domain. To the best of our knowledge, this transfer task (Cityscapes → Indian Vehicles) is the first of its kind. Both are real datasets and we consider five categories including car, truck, bus, motorcycle, and bicycle from both datasets for our experiments. Table 7 shows the results of our method with Faster R-CNN and SSD object detector models and compares them with Faster R-CNN only. It can be seen that we obtain a significant improvement in the performance of the proposed object detector with Faster R-CNN and SSD by 12.5% and 14.7%, respectively. This proves that our work outperforms the above domain adaptation challenge and reduces the distance between the distributions of the two domains. Output images are illustrated in Figure 8b.

### 4.4. Feature Visualization

**Figure 9:**
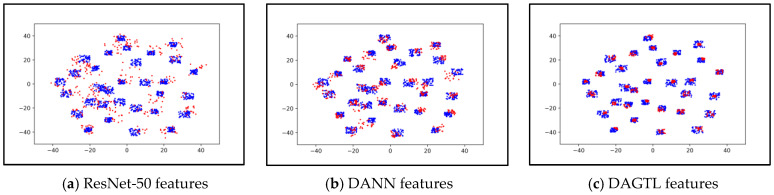
Feature visualization using t-SNE of task A → W of Office-31 dataset. Blue points and red points indicate source and target samples.

To verify the feature transferability of the proposed DAGTL approaches, we utilize t-Distributed Stochastic Neighbor Embedding (t-SNE) [60] to visualize the learned feature embedding of the A → W task. Features are taken from the bottleneck layer after the convergence of the A → W task. ResNet-50 features, DANN features, and DAGTL-IC features are plotted in Figure 9—(a), (b), and (c), respectively. Figure 9a represents the source and target features under the source-only setting, and it is observed that target features are not aligned, indicating a need for domain adaptation. In Figure 9b, DANN discriminates between the source and target features, but there is still a distance between the two domain features. Our proposed DAGTL algorithm aligns the source and target samples the most, as shown in Figure 9c, and shows better intra-class separation and intra-class clusters. This indicates that DAGTL is capable of learning more transferable features, enabling it to effectively adapt to new domains with better feature discrimination.

### 4.5. Parameter Sensitivity and Convergence

**Figure 10:**
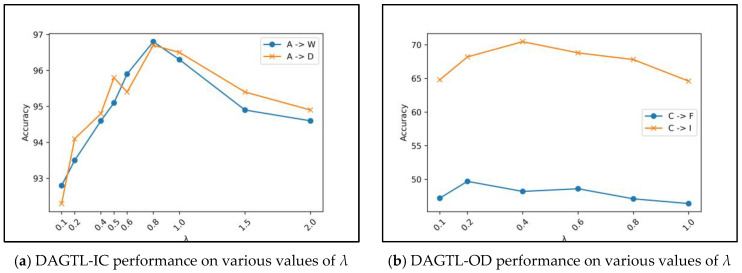
DAGTL performance on various values of *λ*.

We investigate the sensitivity of the parameter *λ* on classification tasks A → W and A → D and object detection tasks C → F and C → I. Figure 10a,b illustrate the classification and object detection performance of these transfer tasks by considering *λ* ∈ {0.1, 0.2, 0.4, 0.5, 0.6, 0.8, 1, 1.5, 2} for classification and *λ* ∈ {0.1, 0.2, 0.4, 0.6, 0.8, 1} for object detection. The trade-off parameter *λ* balances the contributions of domain discriminative loss in the overall objective function which leads to obtaining more discriminating features. Figure 10 reveals that the accuracy increases gradually until *λ* = 0.8 for classification and *λ* = 0.2 and 0.4 for object detection, then accuracy decreases as *λ* increases, following a bell-shaped curve. This demonstrates the effectiveness of *λ* in joint training of learning the classification task and discriminative features for domain adaptation which improves the feature transferability.

**Figure 11:**
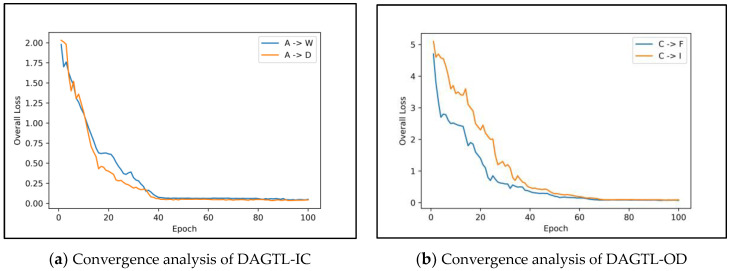
Convergence analysis of DAGTL.

Figure 11a,b depict the convergence analysis of DAGTL-IC on transfer tasks A → W and A → D and DAGTL-OD on transfer tasks C → F and C → I with Faster R-CNN. The convergence graph shows that DAGTL models can stabilize after some iterations. It proves that DAGTL models are superior for cross-domain training with the combined loss of classification or detection loss and domain discriminative loss. Moreover, DAGTL methods converge in fewer iterations compared to adversarial-based approaches [23,28,32,52,53] where specific adversarial network training is required to learn transferable and discriminative feature representations.

### 4.6. Ablation Studies

In this section, ablation experiments are demonstrated to investigate the impact of individual components of the proposed model on overall performance. DAGTL-IC consists of three components with the ResNet-50 network: domain adaptation loss, GTL strategy, and weighted classification loss. We conduct a comprehensive evaluation of DAGTL-IC on a subset of tasks from the Office-31 and Office-Home datasets, and Table 8 presents the accuracy achieved by adding each component for image classification. These tasks have been selected from Table 3, which has a lower transferability score compared to other tasks, to show the performance of the proposed model when there is a considerable domain gap. DAGTL-OD comprises four components with a Faster R-CNN network: image-level discrepancy loss, object-level discrepancy loss, GTL strategy, and weighted classification loss. Table 9 illustrates the contribution of the components of DAGTL-OD in mAP for improving detection performance. The results in Table 8 and Table 9 reveal that each component has some significance for enhancing the overall performance. Results are substantially improved using the guided transfer learning strategy compared to the algorithm with only a domain adaptation loss function. It can also be seen that weighted cross-entropy (classification) loss shows marginal improvement in increasing the overall results.

## 5. Conclusions and Future Work

In this paper, we propose a novel unified unsupervised domain adaptation network to tackle feature transferability during fine-tuning and to align the source and target domain distributions simultaneously for image classification and object detection tasks. We introduce the layer selection strategy using the guided transfer learning approach to fine-tune the model for better knowledge transfer between source and target domains. Furthermore, we employ the JS-Divergence to reduce the domain discrepancy between the domains, which can obtain the domain-invariant features and align the domain distribution. Our proposed UDA networks utilize the ResNet-50 network as a backbone. Extensive experimental analysis reveals that our proposed method has the ability to learn the domain invariant feature representations by training the algorithm using the layer selection strategy and domain discrepancy loss. It is also observed in the ablation study that our method has an obvious advantage in learning more transferable features based on the similarity score between the domains using the layer selection strategy. It is also important to note that training of our objective function is based on domain discrepancy loss, thus it requires less convergence time compared to adversarial-based approaches. The DAGTL-IC approach improves accuracy by 1.8% and 2.6% on Office-31 and Office-Home datasets, respectively compared to the state-of-the-art method. Similarly, the DAGTL-OD approach achieves 4.3% and 14.7% improvements in mAP on Foggy Cityscapes and the Indian vehicle dataset, respectively. These results demonstrate the effectiveness of our approach for domain adaptive image classification and object detection.

In the future, the proposed approaches can be applied to various real-world applications where improving performance through transferability and domain alignment between source and target domains are primary concerns. Furthermore, these approaches can be extended to other deep CNN backbone networks to enhance the performance of image classification and real-time object detection.

## Figures and Tables

**Table 1:** Comparative summary of the existing domain adaptive image classification methods.

**Method**	**Type of Domain Adaptation**	**Base Network**	**Loss**	**Datasets**			**Year**
				**Office-31** [37]	**Office-Home** [38]	**Digits** **(MNIST** [39]/**USPS** [40])	
DDC [5]	Discrepancy-based	AlexNet	MMD	✓	-	-	2014
DAN [13]	Discrepancy-based	AlexNet	MK-MMD	✓	-	-	2015
DANN [23]	Adversarial-based	AlexNet	GAN-based Discriminator	✓	-	✓	2015
CORAL [17]	Discrepancy-based	AlexNet	CORAL	✓	-	-	2016
ADDA [24]	Adversarial-based	AlexNet & ResNet-50	GAN-based Discriminator	✓	-	✓	2017
JAN [14]	Discrepancy-based	ResNet-50	JMMD	✓	-	-	2017
CDAN [35]	Discrepancy-based	ResNet-50	Conditional- based Discriminator	✓	✓	✓	2018
MADA [33]	Adversarial-based	ResNet-50	GAN-based Discriminator	✓	-	-	2018
SimNets [34]	Adversarial-based	ResNet-50	GAN-based Discriminator	✓	-	✓	2018
CAN [7]	Discrepancy-based	ResNet-50	CCD	✓	-	-	2019
SymNets [28]	Adversarial-based	ResNet-50	GAN-based domain confusion	✓	✓	-	2019
SGC [19]	Discrepancy-based	ResNet-50	JMMD	✓	✓	✓	2020
MDDA [22]	Discrepancy-based	ResNet-50	MMD	✓	✓	✓	2020
HAN [30]	Discrepancy & Adversarial-based	ResNet-50	CORAL and GAN-based Discriminator	✓	✓	-	2020
GSDA [29]	Adversarial-based	ResNet-50	Global and local Adversarial Discriminator	✓	✓	-	2020
SRDC [31]	Adversarial-based	ResNet-50	Clustering-based Discriminator	✓	✓	-	2020
FixBi [32]	Adversarial-based	ResNet-50	Augmentation	✓	✓	-	2021
CAF [21]	Discrepancy-based	ResNet-50	Wasserstein distance	✓	-	-	2022
DALN [36]	Adversarial-based	ResNet-50	NWD-based Discriminator	✓	✓	-	2022

**Table 2:** Comparative summary of the existing domain adaptive object detection methods.

**Method**	**Detection Network**	**Loss**	**Datasets**			**Year**
			**Cityscapes** [58]	**Foggy Cityscapes** [58]	**KITTI** [59]	
DA-Faster [46]	Faster R-CNN	H-divergence based Discriminator	✓	✓	✓	2018
SWDA [47]	Faster R-CNN	Weak Global and Strong local Feature Alignment	✓	✓	✓	2019
SCDA [48]	Faster R-CNN	Region-Level Adversarial Alignment	✓	✓	-	2019
CFA [49]	Faster R-CNN	Prototype-based Semantic Alignment	✓	✓	✓	2020
MAF [50]	Faster R-CNN	Adversarial domain alignment loss	✓	✓	✓	2019
CDN [52]	Faster R-CNN	CDN-based adversarial loss	✓	✓	✓	2020
HTCN [53]	Faster R-CNN	Pixel-wise adversarial loss	✓	✓	-	2020
ODSC [54]	SSD	Pseudo Labels and Style Transfer alignment	✓	✓	-	2020
SFA [55]	DefDETR	Token-wise and Hierarchical Sequence Feature Alignment loss	✓	✓	-	2021
MGA [56]	Faster R-CNN & FCOS	Pixel-level, instance-level, and category-level.	✓	✓	✓	2022
O^2^net [57]	DefDETR	Pixel- and instance-level	✓	✓	-	2022

**Table 3:** Transferability score and *k*th layer ($L_{k}$ ) between the source and target domains.

Domains	A & W	A & D	W & D	Ar & Cl	Ar & Pr	Ar & Rw	Cl & Pr	Cl & Rw	Pr & Rw	C & F	C & I
τ−score	0.758	0.721	0.93	0.65	0.72	0.775	0.7	0.68	0.836	0.88	0.67
$L_{k}$	37	35	45	31	35	38	34	33	41	43	32

**Table 4:** Result Analysis of the Office-31 dataset.

Methods (Source → Target)	A → D	A → W	D → A	D → W	W → A	W → D	Avg. Accuracy
ResNet-50	68.9	68.4	62.5	96.7	60.7	99.3	76.1
CORAL [17]	81.5	77.0	65.9	97.1	64.3	99.6	80.9
DANN [23]	79.7	82.0	68.2	96.9	67.4	99.1	82.2
ADDA [24]	77.8	86.2	69.5	96.2	68.9	98.4	82.9
JAN [14]	84.7	85.4	68.6	97.4	70.0	99.8	84.3
MADA [33]	87.8	90.0	70.3	97.4	66.4	100	85.2
MDDA [22]	86.3	86.0	72.1	97.1	73.2	99.2	85.7
SimNets [34]	88.6	85.3	73.4	98.2	71.8	99.7	86.2
SymNets [28]	93.9	90.8	74.6	98.8	72.5	100	88.4
HAN [30]	95.3	94.4	72.1	98.8	71.7	100	88.7
GSDA [29]	94.8	95.7	73.5	99.1	74.9	100	89.7
SRDC [31]	95.8	95.7	76.7	99.2	77.1	100	90.8
DALN [36]	95.4	95.8	76.4	99.1	76.5	100	90.4
FixBi [32]	95.0	96.1	78.7	99.3	79.4	100	91.4
Ours (DAGTL-IC)	97.2	97.1	82.9	99.2	82.7	100	93.2

**Table 5:** Result Analysis of the Office-Home dataset.

Source ↓ Target	Ar ↓ Cl	Ar ↓ Pr	Ar ↓ Rw	Cl ↓ Ar	Cl ↓ Pr	Cl ↓ Rw	Pr ↓ Ar	Pr ↓ Cl	Pr ↓ Rw	Rw ↓ Ar	Rw ↓ Cl	Rw ↓ Pr	Avg. Accuracy
ResNet-50	34.9	50.0	58.0	37.4	41.9	46.2	38.5	31.2	60.4	53.9	41.2	59.9	46.1
CORAL [17]	42.2	59.1	64.9	46.4	56.3	58.3	45.4	41.2	68.5	60.1	48.2	73.1	55.3
DANN [23]	45.6	59.3	70.1	47.0	58.5	60.9	46.1	43.7	68.5	63.2	51.8	76.8	57.6
JAN [14]	45.9	61.2	68.9	50.4	59.7	61.0	45.8	43.4	70.3	63.9	52.4	76.8	58.3
CDAN [35]	46.6	65.9	73.4	55.7	62.7	64.2	51.8	49.1	74.5	68.2	56.9	80.7	62.8
MDDA [22]	54.9	75.9	77.2	58.1	73.3	71.5	59.0	52.6	77.8	67.9	57.6	81.8	67.3
SymNets [28]	47.7	72.9	78.5	64.2	71.3	74.2	63.6	47.6	79.4	73.8	50.8	82.6	67.2
GSDA [29]	61.3	76.1	79.4	65.4	73.3	74.3	65.0	53.2	80.0	72.2	60.6	83.1	70.3
SRDC [31]	52.3	76.3	81.0	69.5	76.2	78.0	68.7	53.8	81.7	76.3	57.1	85.0	71.3
DALN [36]	57.8	79.9	82.0	66.3	76.2	77.2	66.7	55.5	81.3	73.5	60.4	85.3	71.8
FixBi [32]	58.1	77.3	80.4	67.7	79.5	78.1	65.8	57.9	81.7	76.4	62.9	86.7	72.7
Ours (DAGTL-IC)	61.3	80.5	83.2	70.2	82.5	80.4	69.2	61.8	84.1	75.8	65.1	89.5	75.3

**Table 6:** Result analysis from Cityscapes to Foggy-Cityscapes domain adaptation.

Methods	Person	Rider	Car	Truck	Bus	Train	Mcycle	Bicycle	mAP
Faster R-CNN	17.8	23.6	27.1	11.9	23.8	9.1	14.4	22.8	18.8
DA-Faster [46]	25.0	31.0	40.5	22.1	35.3	20.2	20.1	27.1	27.6
SCDA [48]	33.5	38.0	48.5	26.5	39.0	23.3	28.0	33.6	33.8
ODSC [54]	29.9	42.3	43.5	24.5	36.2	32.6	35.3	30.0	34.3
SWDA [47]	30.3	42.5	44.6	24.5	36.7	31.6	30.2	35.8	34.8
CDN [52]	35.8	45.7	50.9	30.1	42.5	29.8	30.8	36.5	36.6
HTCN [53]	33.2	47.5	47.9	31.6	47.4	40.9	32.3	37.1	39.8
SFA [55]	46.5	48.6	62.6	25.1	46.2	29.4	28.3	44.0	41.3
MGA [56]	43.9	49.6	60.6	29.6	50.7	39.0	38.3	42.8	44.3
O^2^net [57]	48.7	51.5	63.6	31.1	47.6	47.8	38.0	45.9	46.8
Ours (FRCNN)	50.2	52.2	63.5	36.7	57.5	47.8	40.6	49.8	49.7
Ours (SSD)	51.8	51.4	62.2	38.4	63.1	49.8	38.8	53.4	51.1

**Table 7:** Result analysis from Cityscapes to Indian Vehicle dataset domain adaptation.

Methods	Car	Truck	Bus	Mcycle	Bicycle	mAP
Faster R-CNN	70.8	48.6	50.3	65.2	55.3	58.0
Ours (FRCNN)	85.8	61.3	65.4	78.5	61.3	70.5
Ours (SSD)	82.5	65.9	69.7	82.5	63.1	72.7

**Table 8:** Ablation results by the components of the proposed DAGTL-IC approach on Office-31 and Office-Home in accuracy.

	ResNet-50	$\mathrm{ResNet}-50+L_{d}$	$\mathrm{ResNet}-50+L_{d}+\mathrm{GTL}$	$\mathrm{ResNet}-50+L_{d}$ $+\mathrm{GTL}+\mathrm{Weighted}\;\mathrm{Classification}\;\mathrm{Loss}\;(L_{c})$	A → W	A → D	W → A	D → A	Ar → Rw	Cl → Pr
ResNet-50	✓				68.4	68.9	60.7	62.5	58	41.9
Proposed Model	✓	✓			95.6	95.4	77.3	77.6	78.9	77.8
	✓	✓	✓		96.2	96.4	81.1	80.1	81.3	80.5
	✓	✓	✓	✓	97.1	97.2	82.7	82.9	83.2	82.5

**Table 9:** Ablation results by the components of the proposed DAGTL-OD approach on Foggy cityscapes and Indian Vehicle dataset in mAP.

	Faster R-CNN	$\mathrm{Faster}\;R-\mathrm{CNN}+L_{img}$	Faster R-CNN $+L_{obj}$	Faster R-CNN $+L_{img}$ $+L_{obj}$ + GTL	$\mathrm{Faster}\;R-\mathrm{CNN}+L_{img}$ $+L_{obj}$$+\mathrm{GTL}+\mathrm{Weighted}\;\mathrm{Classification}\;\mathrm{Loss}\;(L_{cls})$	C → F	C → I
Faster R-CNN	✓					18.8	58.0
Proposed Model	✓	✓				44.6	63.5
	✓	✓	✓			46.2	66.9
	✓	✓	✓	✓		48.9	69.2
	✓	✓	✓	✓	✓	49.7	70.5

## Data Availability

Data available in a publicly accessible repository that does not issue DOIs. Office-31, Office-Home, Cityscapes and Foggy Cityscapes are publicly available datasets and were analyzed in this study. These data can be found here: [https://faculty.cc.gatech.edu/~judy/domainadapt/] (accessed on 10 November 2022), [https://www.hemanthdv.org/officeHomeDataset.html] (accessed on 15 November 2022) and [https://www.cityscapes-dataset.com/] (accessed on 20 November 2022). The Indian Vehicle dataset was obtained from Vadodara Smart City Development Limited (VSCDL), City Command & Control Centre, Vadodara and is available from the authors with the permission of VSCDL.
